# Dorsal venous complex ligation‐free and parietal endopelvic fascia preserving in laparoscopic radical prostatectomy: A prospective study of single centre

**DOI:** 10.1002/bco2.437

**Published:** 2024-09-19

**Authors:** Zhong‐Hua Yang, Yong‐Zhi Wang, Tao Liu, Hang Zheng, Xing‐Huan Wang

**Affiliations:** ^1^ Department of Urology Zhongnan Hospital of Wuhan University Wuhan Hubei China

**Keywords:** dorsal venous complex, endopelvic fascia, parietal, prostate cancer, radical prostatectomy

## Abstract

**Objectives:**

This study aims to describe a novel dorsal venous complex (DVC) ligation‐free and parietal endopelvic fascia preserving technique for laparoscopic radical prostatectomy and to evaluate its post‐operative outcomes.

**Methods:**

From April 2020 to May 2021, a total of 125 patients with localized prostate cancer received laparoscopic radical prostatectomy by a single surgeon. In the procedure, a novel technique of DVC ligation‐free and parietal endopelvic fascia preserving was used. Preoperative characteristics of patients and perioperative results were recorded. In this study, continence was defined as zero to one pad per day. Oncological outcomes were evaluated based on positive surgical margin.

**Results:**

Five patients required a blood transfusion. Mean post‐operative hospital stay was 3.9 days (2–5), and the catheter could be removed on post‐operative day 7 to 9. Final pathologic evaluations were 87 stage pT2, 22 stage pT3a, and 7 pT3b, 9 stage pT4, respectively. The positive surgical margin rate was 10.4% in total. Ninety‐three patients (74.4%) returned to urinary continence 2 months post‐operatively, and 11 patients (11/125) developed biochemical recurrence 6 months post‐operatively.

**Conclusions:**

The DVC ligation‐free and parietal endopelvic fascia preserving technique provides early recovery from incontinence without adversely affecting the oncological outcome.

## 1 INTRODUCTION

The dorsal venous complex (DVC) is located ventrally of the prostate and urethral sphincter containing the dorsal vein complex draining the blood of penile veins and small arteries originating from the inferior vesical artery. Transecting the DVC and puboprostatic ligaments (PPL) without ligation can cause substantial bleeding, especially in open operation. However, injury to the urethral sphincter may occur easily when ligating the DVC, translating into potential post‐operative urinary incontinence.^1^ On the other hand, the pelvic fascia can be divided into a parietal and a visceral endopelvic fascia. Dissection the parietal endopelvic fascia has been thought to be a routine procedure to make the prostate of most mobility. However, incision of the parietal endopelvic fascia may injury the underlying levator ani muscle (LAM) directly or cause LAM exposed to the urine or inflammatory exudation and impaired indirectly. It is reported that the reduced LAM contraction might not compress the urethra sufficiently when abdominal pressure raised, which plays an important role in post‐operative urinary incontinence.^2^

This study aimed to describe the key surgical steps of DVC ligation‐free and parietal endopelvic fascia preserving in laparoscopic prostatectomy and explore its impact on intraoperative blood loss, incidence of positive surgical margin and urinary continence of the patient.

## 2 SURGICAL TECHNIQUE

Subjects were positioned in the supine position with both arms tucked. A 3 cm infra‐umbilical vertical incision is made one finger breadth below the umbilicus. The anterior sheath of rectus abdominis is incised transversely and blunt dissection is performed using the index finger between the rectus abdominis and posterior sheath of rectus abdominis. After the initial development of extra‐peritoneal space, four trocars were placed.

**FIGURE 1:**
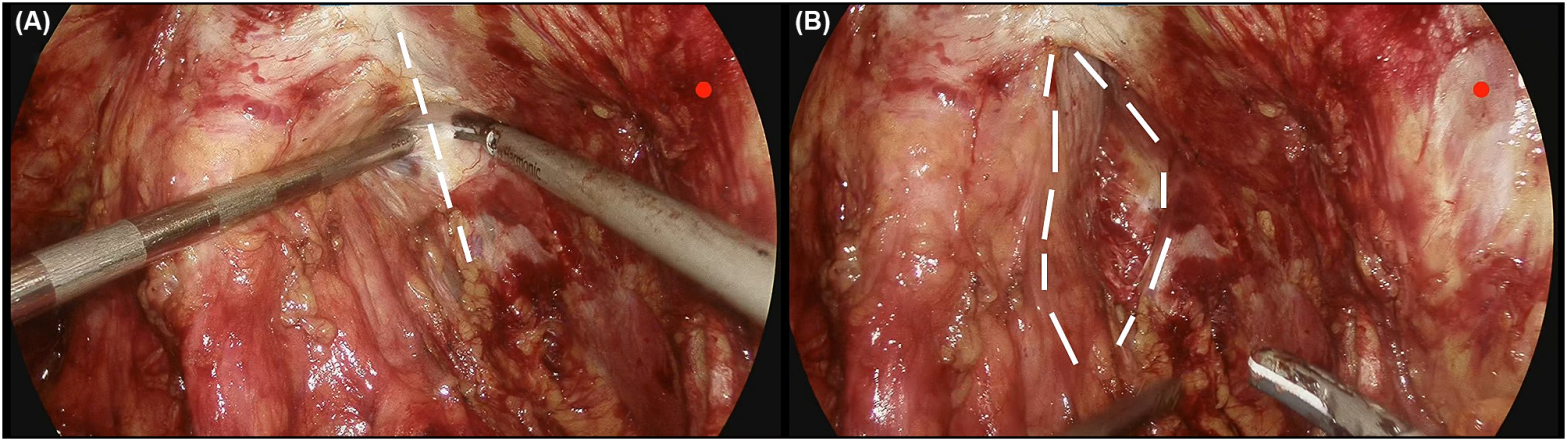
(A) The parietal and visceral components of the endopelvic fascia are bluntly separated along the fascial tendinous arch of the pelvis where those two parts fused so as to preserve the parietal endopelvic fascia (showing with the white dashed line). (B) The bluntly separated parietal and visceral components of the endopelvic fascia. The white dashed lines show the detached fascial tendinous arch of the pelvis.

After extended pelvic lymphadenectomy, if needed, and removal of the periprostatic fat, the endopelvic fascia is exposed. The parietal and visceral components of the endopelvic fascia are bluntly separated along the fascial tendinous arch of the pelvis where those two parts fused so as to preserve the parietal endopelvic fascia (Figure 1).

**FIGURE 2:**
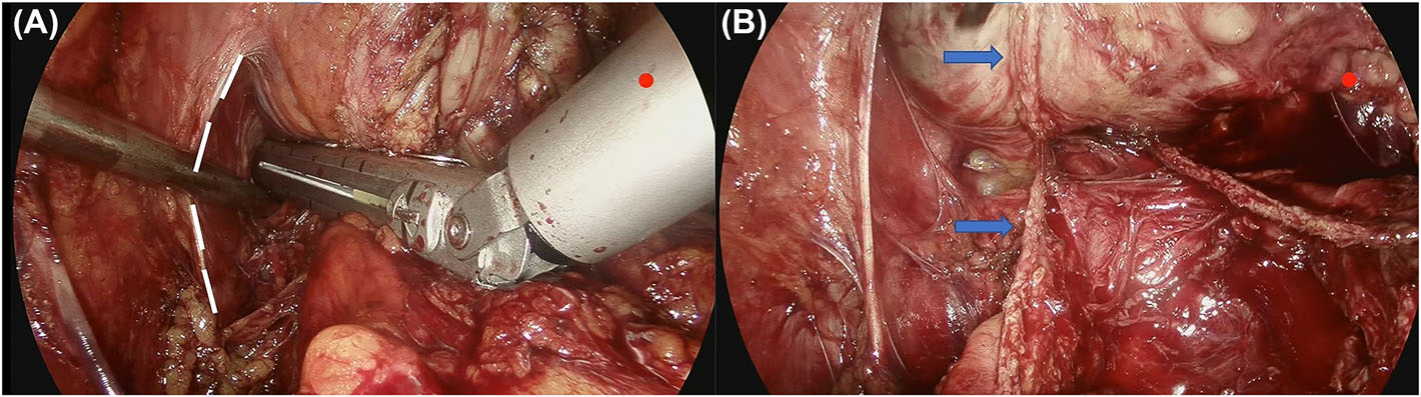
(A) The left prostatic pedicle is divided with an automatic excision anastomosis device after Denonvilliers' fascia incision and posterior prostatic surface dissection. The white dashed lines show the detached ascial tendinous arch of the pelvis. (B) The divided left prostatic pedicle (blue arrows).

Following the division of the anterior and posterior aspects of the bladder neck, both vasa and seminal vesicles were exposed and dissected as standard procedure. After Denonvilliers' fascia incision and posterior prostatic surface dissection, the prostatic pedicles are divided with an automatic excision anastomosis device (Echelon Flex) (Figure 2).

**FIGURE 3:**
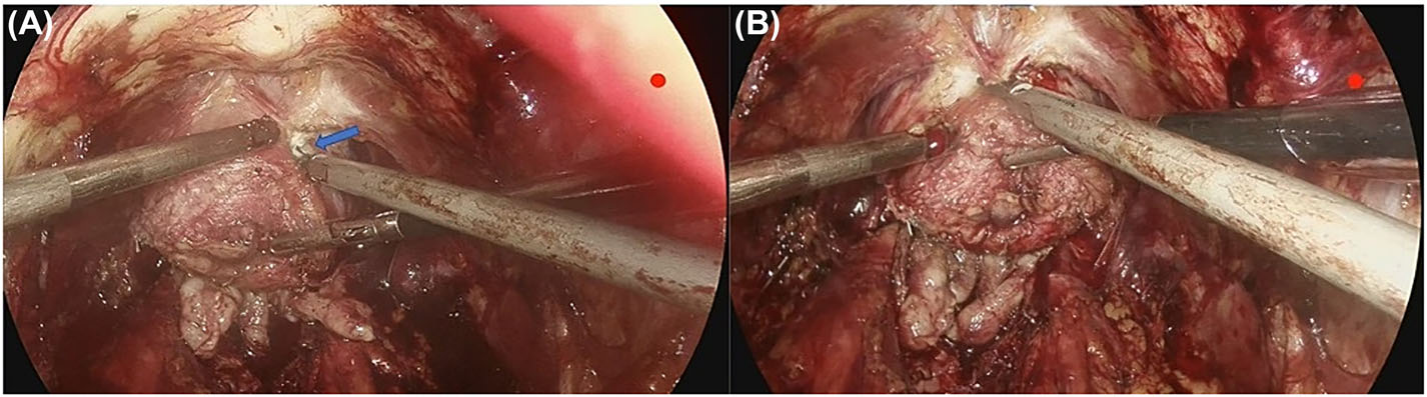
The detrusor apron is transected where the puboprostatic ligaments fusion with the detrusor apron, or near the base of the prostate with a Harmonic, and the periprostatic fascia, including the DVC, detrusor apron and puboprostatic ligaments were dissected from the ventral surface of the prostate below the plane of the vessel without substantial bleeding.

To prevent bleeding from the unligated DVC, the detrusor apron (DA) is transected where the PPL fusion with the DA, or near the base of the prostate with a Harmonic (ETHICON GEN11), where the diameters of the DVC is much smaller than those at the apex (Figure 3). Advance along the cleavage plane between the periprostatic fascia and DA, DA and PPL were dissected from the ventral surface of prostate without substantial bleeding. Once the beginning of the membranous urethra was reached, the urethra can be divided sharply, and the prostate was completely detached. Finally, the urethro‐vesical anastomosis is completed with running suture using 2/0 double‐ended barbed suture (Stratafix; Ethicon) over a 22‐Fr catheter.

The study was approved by the Ethical Committee of Wuhan University School of Medicine.

## 3 DATA ACQUISITION

Perioperative characteristics including age, clinical stage, prostate‐specific antigen (PSA) level and pathological and surgical variables were collected.

## 4 RESULTS

All the 125 patients received the operation and completed the 6‐month follow‐up. As demonstrated in Table 1, the mean age was 67.4 years with a mean PSA level at operation 15.5 ng/mL. The mean volume of prostate is 33.6 mL. About a half of patients were classified as intermediate risk by PSA level and clinical stage. Sixty‐nine of the patients were cT2 and 47 were cT1c, respectively.

**TABLE 1:** Preoperative patient characteristics.

Parameters	Value, mean (range)
Age, yr (range)	67.4 (57–73)
Mean PSA, ng/mL (range)	15.5 (4.4–35.5)
TRUS prostate volume, mL	33.6 (21–77)
Gleason score of the biopsy	
3 + 3	45
3 + 4, 4 + 3	67
3 + 5, 4 + 4, 5 + 3	11
4 + 5, 5 + 4, 5 + 5	2
Clinical stage	
cT1c	47
cT2a, cT2b, cT2c	69
cT3a, cT3b	9
Comorbidities	
Hypertension	11
DM	7

Perioperative and post‐operative data were demonstrated in Table 2. The mean operative time is 84.4 min (ranging from 65 to 145 min), and blood loss was 105 mL, while five received transfusion for bleeding more than 400 mL. The patients were dismissed from hospital 3.9 days post‐operatively, and the bladder catheters were removed 7.4 days post‐operatively, ranging from 7 to 9 days. As for the pathological staging, 87 patients were classified as pT2 and nine as pT4, and 13 patients were diagnosed as positive surgical margin. All the nine patients of pT4 stage have positive surgical margins, of which three patients having more than two positive surgical margins. One patient of pT2 stage harbouring a positive margin located at the apex of prostate dorsally. Three cases with pT3 stage demonstrated one to two positive surgical margins, of which one margin located at the apex of prostate ventrally. Ninety‐thre patients returned urinary continence in 2 months post‐operatively, while 11 patients experienced biochemical recurrence in 6 months post‐operatively.

**TABLE 2:** Perioperative and post‐operative data.

Parameters	Value, mean (range)
Operative time, min (range)	85.4 (65–145)
Transfusion rate	5/125 (4%)
Blood loss, mL	105 (50–550)
Bladder catheterization time, d	7.4 (7–9)
Urinary retention	0/125 (0)
Stenosis of the anastomosis	0/125 (0)
Hospital stay post‐operatively, d	3.9 (2–5)
Pathologic stage	
pT2	87
pT3a	22
pT3b	7
pT4	9
Positive surgical margin	13
Continence at 2 months post‐operatively	93/125 (74.4%)
Biochemical recurrence 6 months post‐operatively	11/125

## 5 DISCUSSION

Urinary incontinence (UI) after radical prostatectomy (RP) is a common side effect with a major impact on many aspects of their lives.^3^ With the increasing number of RP, the impact of UI is becoming an increasing problem that requires an optimal approach to its prevention and treatment. The UI after RP is influenced by multiple elements, including anatomic components.^4^ The anatomic components influencing urinary continence, after RP, are the urethral sphincter complex, the supporting structures of the membranous urethra and^5^ the fibrosis after surgery.^6^

As a routine procedure, an incision of the parietal endopelvic fascia is performed and then carefully extended in an anteromedial direction towards the PPL. This allows the surgeon to palpate the lateral surface of the prostate. On the other hand, however, this may injury the underlying LAM directly or cause LAM exposed to the urine or inflammatory exudation and impaired indirectly. Shin et al. found that the reduced muscle contraction might not compress the urethra sufficiently when abdominal pressure is raised.^2^ In our series, the two lays of endopelvic fascia, means visceral and parietal endopelvic fascia, were bluntly separated, and the LAM was kept from exposure to urine or inflammatory exudation.

As for the ligation of DVC, in the traditional or robotic‐assisted laparoscopic technique, due to the increased pressure of pneumoperitoneum, blood loss was not found to be significantly different no matter DVC ligation was used or not.^7^ In our series, the DA is transected where the PPL fusion with the DA, or near the base of the prostate with a Harmonic, rather than transecting the DVC at the apex of the prostate, and the periprostatic fascia, including the DVC, DA and PPL were dissected from the ventral surface of the prostate below the plane of the vessel without substantial bleeding. Several non‐randomized, prospective or retrospective studies supported the technique of ligation‐free for higher and faster continence after radical prostatectomy^8^, ^9^, ^10^ (Table 3). While the only randomized controlled study by Carlo et al. demonstrated that DVC ligation or not did not affect recovery of urinary continence after surgery.^11^

**TABLE 3:** Studies assessing effect of DVC ligation‐free on continence recovery.

Study	*n*	Design	Follow‐up	Treatment	Effect on urinary continence and bleeding
Bravi et al.^11^	216	Randomized controlled	4 months	DVC ligation versus ligation‐free	Urinary continence, 24% versus 29% (1 month); 72% versus 65% (4 months) (both *p* = 0.3)
Cheng et al.^8^	154	Non‐randomized controlled	3 months	DVC ligation versus ligation‐free	Urinary incontinence, 2.5% versus 17.5%, *p* < 0.05; blood loss (ΔZ: [2.11 ± 8.88] vs. [1.24 ± 14.70] g/L, *p* > 0.05)
Liu et al.^9^	314	Retrospective study	6 months	DVC ligation versus ligation‐free	Higher and faster continence recovery in the Comb‐RP group (mean 4.9 vs. 2.6 months, log rank *p* = 0.001)
Porpiglia et al.^10^	60	Prospective randomized	12 months	DVC ligation versus ligation‐free	A higher continence rate in ligation‐free group after 3 months: 53% versus 80%, *p* < 0.05

## 6 CONCLUSIONS

Herein, we provided and described a novel DVC ligation‐free and parietal endopelvic fascia preserving technique for laparoscopic radical prostatectomy. It is safe and feasible. We use the technique even if there is a disease at the apex of the prostate. However, in rare cases of a radiologically suspicious and/or histologically confirmed lesion in the anterior part of prostate, we ligate and divide the DVC at the apex of the prostate, without preserving the PPL and DA. Because it is thought that in anterior part of prostate, there is no loose fascia between the prostate capsule and DA in anterior part of prostate, and preserving the detrusor apron may result in a positive margin in the anterior prostate. However, for this is a prospective and observational study, we did not compare the perioperative characteristics or oncologic or functional results with conventional technique, and a further control trial may be needed to evaluate the efficacy of this technique.

## AUTHOR CONTRIBUTIONS

Xing‐Huan Wang and Hang Zheng conceived the idea and its design. Yong‐Zhi Wang and Tao Liu collected the data and performed data analyses. Zhong‐Hua Yang performed the surgical procedure and drafted the manuscript. All authors contributed to the article and approved the submitted version. All authors read and approved the final manuscript.

## CONFLICT OF INTEREST STATEMENT

The authors declare no conflicts of interest.
